# Variant calling in low-coverage whole genome sequencing of a Native American population sample

**DOI:** 10.1186/1471-2164-15-85

**Published:** 2014-01-30

**Authors:** Chris Bizon, Michael Spiegel, Scott A Chasse, Ian R Gizer, Yun Li, Ewa P Malc, Piotr A Mieczkowski, Josh K Sailsbery, Xiaoshu Wang, Cindy L Ehlers, Kirk C Wilhelmsen

**Affiliations:** 1 Renaissance Computing Institute, University of North Carolina at Chapel Hill, Chapel Hill, USA; 2 Department of Genetics, University of North Carolina at Chapel Hill, Chapel Hill, USA; 3 Department of Psychological Sciences, University of Missouri-Columbia, Columbia, USA; 4 UNC High Throughput Sequencing Facility, Chapel Hill, USA; 5 Department of Molecular and Cellular Neuroscience, The Scripps Research Institute, La Jolla, USA

## Abstract

**Background:**

The reduction in the cost of sequencing a human genome has led to the use of genotype sampling strategies in order to impute and infer the presence of sequence variants that can then be tested for associations with traits of interest. Low-coverage Whole Genome Sequencing (WGS) is a sampling strategy that overcomes some of the deficiencies seen in fixed content SNP array studies. Linkage-disequilibrium (LD) aware variant callers, such as the program Thunder, may provide a calling rate and accuracy that makes a low-coverage sequencing strategy viable.

**Results:**

We examined the performance of an LD-aware variant calling strategy in a population of 708 low-coverage whole genome sequences from a community sample of Native Americans. We assessed variant calling through a comparison of the sequencing results to genotypes measured in 641 of the same subjects using a fixed content first generation exome array. The comparison was made using the variant calling routines GATK Unified Genotyper program and the LD-aware variant caller Thunder. Thunder was found to improve concordance in a coverage dependent fashion, while correctly calling nearly all of the common variants as well as a high percentage of the rare variants present in the sample.

**Conclusions:**

Low-coverage WGS is a strategy that appears to collect genetic information intermediate in scope between fixed content genotyping arrays and deep-coverage WGS. Our data suggests that low-coverage WGS is a viable strategy with a greater chance of discovering novel variants and associations than fixed content arrays for large sample association analyses.

## Background

As compared to whole genome sequencing (WGS), at the present time, genotype sampling is a more cost effective strategy to identify variants associated with traits of interest. Genome-wide association studies (GWAS) using fixed content marker arrays represent a genotype sampling strategy that has been successfully used in a number of studies to identify SNPs significantly associated with complex traits [1-3]. Additionally, by increasing the number of markers genotyped and using linkage disequilibrium to guide marker selection an increasing proportion of genomic variance can be interrogated during GWAS [3]. The principal caveat of using fixed content arrays is that there is a diminishing return on investment for adding additional markers and a portion of the sequence variation is expected to be poorly interrogated. Further, variants that are specific to any population that are not used to guide SNP selection, including variants that are specific to a particular trait, are likely to be poorly interrogated. Finally, trait-specific and rare variants may be poorly imputed from the typed markers. Identifying population-specific sequence variants and adding them to the fixed content is a potential solution to this problem in order to increase the effectiveness of GWAS. However, if sequencing costs continue to decline, it will eventually become more effective to employ sequencing techniques using the population of interest to find trait-associated variants.

Even with exponential declines in the cost of next-generation genomic sequencing, there are still difficulties associated with using WGS to conduct association studies of complex traits because the moderate to small effect sizes of variants typically involved in the etiology of such traits requires large sample sizes. One approach to increase the power of such studies without increasing sequencing cost is to use whole-exome sequencing (WES), in which only a small fraction of the genome is sequenced, but at high coverage (i.e., high average number of sequence reads for a given base pair) [4,5]. A second approach is to perform WGS, but reduce the overall coverage. The success of this low-coverage strategy is contingent upon the ability to locate variant sites and accurately call genotypes when each site may only be covered by a small number of reads (e.g., less than 5× coverage). However, if variant calling in low-coverage WGS is acceptable, the increased genomic landscape sequenced relative to GWAS and WES allows for a greater chance of discovering novel variants and associations.

The variant calling algorithms that are currently used to measure genotypes from sequence data can be divided into three groups, each using successively more information. The simplest variant callers make use of the reads collected at a single genomic position from a single sample, and apply a statistical model to determine the most likely genotype at that position for that sample. The second type of variant caller includes information from multiple samples at a single site. In this case the multiple samples provide a prior likelihood that the site is a variant, and the caller determines the most likely distribution of alleles given the data. Both of these methods are implemented in the program GATK Unified Genotyper [6,7]. The third type of caller uses multiple samples, but also incorporates the observed correlation between nearby variants, *i.e.* linkage-disequilibrium (LD), to improve calls. The program Thunder, which is based on the MACH imputation algorithm [8], uses a Hidden Markov Model to identify genomic regions shared by different samples, and uses this information in calling variants [9]. In [9], Li *et al.* show that LD-based variant calling using Thunder increases calling accuracy in low-coverage samples to the point at which low-coverage approaches become viable.

Low-coverage WGS produces high quality genotype calls for common polymorphisms (i.e., a minor allele frequency > 5% in the population) where several of the sequenced samples can be expected to have the minor allele. The confidence with which rarer alleles are detected depends on the quality of the sequence, the number of individuals in the sample, and the prior probability that the allele exists. Low-coverage WGS detects more polymorphisms than can possibly be detected with existing fixed content arrays but is not as good as deep WGS for detecting with confidence rare alleles or alleles that are specifically typed with fixed content arrays. The utility of low-coverage WGS will be greatest for studying populations that have not been subjected to prior systematic variant detection. Low-coverage WGS with ~4X coverage currently costs between 1 and 6 fold more per sample than fixed content genotyping arrays, depending on coverage. If the cost of WGS falls, the current relative economic advantage of fixed content genotyping arrays is expected to decline in favor of the more comprehensive WGS.

The current report is part of an ongoing family study to identify sequence variants that predispose to substance dependence in a community sample of American Indians [10-14].

**Figure 1:**
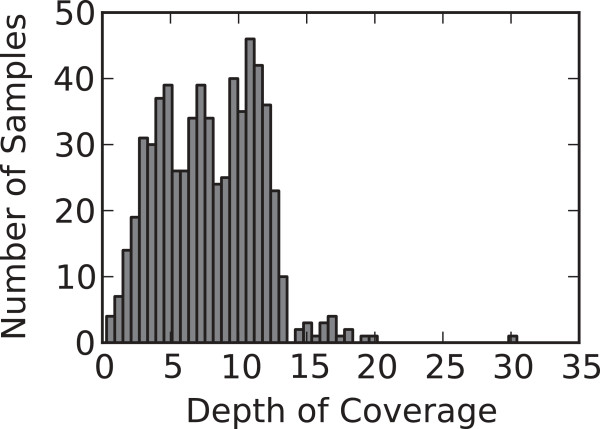
**Sample depth of coverage.** Histogram of the mean sequencing read depth per sample for 641 samples. 88% of the samples have mean depth less than 13, and 26% have depth less than 5.

The specific aims of this report were to (1) validate the low-coverage WGS approach by comparing different variant calling approaches and the resulting variant calls from low-coverage WGS (3-12X see Figure 1) to genotypes for approximately 200,000 polymorphic variants measured with a first generation Axiom Affymetrix Exome Chip array and (2) provide a preliminary demonstration of the utility of the called variant data obtained from low-coverage WGS by investigating the kinship structure and calculating founder allele frequencies for this sample.

## Results and discussion

### Sample concordance

To evaluate the efficacy of variant calling methods the results of each caller were compared to genotypes from the fixed content exome SNP chip. Because the exome chip is specifically constructed to contain low-frequency markers, there is a subset of markers that have not been validated by Affymetrix in known heterozygotes. An initial comparison between exome chip genotypes and Thunder’s variant calls shows that for roughly 0.5% of the non-monomorphic exome chip sites, concordance is essentially zero, indicating a systematic failure of the marker on the exome chip, with A and B alleles becoming reversed. While this data could potentially be recovered by reversing the A and B alleles in analysis, the chip manufacturer recommends dropping this relatively small number of markers. We therefore remove from consideration any marker with a concordance less than 2% across all samples (998 markers).

We define the concordance of sample S as *C*_*S*_ = *N*_*S,m*_*/N*_*S,v*_, where *N*_*S,v*_ is the number of genotypes for sample S that contain at least one non-reference allele on the exome chip, and *N*_*S,m*_ is the number of those genotypes where the variant call matches the exome chip genotype. For the purposes of this calculation, an explicit or implicit missing genotype call from a given caller is interpreted as a homozygous reference call.

**Figure 2:**
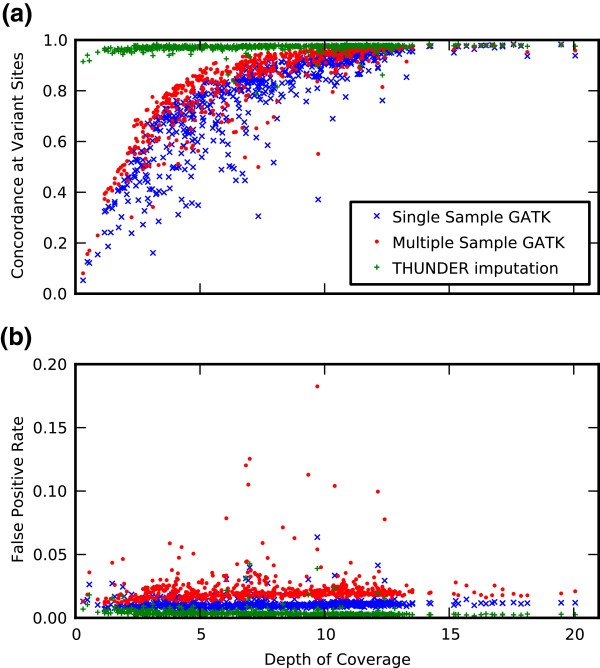
**Concordance with exome chip.** Concordance between exome chip genotypes and genotypes from three variant callers **(a)** and false positive rate **(b)**. One point (at depth = 30.4, concordances between 96.7% and 98%) has been removed to expand the data region. The concordance is calculated only at the sites that are measured as non-monomorphic in the exome chip genotypes.

These calculations revealed a number of notable trends (see Figure 2a). First, increased information included in the calling algorithm leads to an increased fidelity of calling, with multi-sample calling outperforming single sample calling, and LD-aware methods outperforming both. The median concordance rates are 85.5% (single sample), 90.4% (multi-sample), and 97.5% (LD-aware). Second, the marked dependence on sample depth observed with single sample and multi-sample calling is reduced for LD-aware calling. For samples with average coverage less than two, single sample and multi-sample callers fare poorly, with concordances dropping below 10 percent, whereas with Thunder calling, no sample has less than 78.8% concordance. The median improvement of LD-aware calling over the other methods is dependent on depth; for samples with less than 5X coverage, the median improvement is approximately 30%, decreasing to only 2% for samples with greater than 10X coverage. Of note, concordance rates for LD-aware calling were similar in magnitude for variants inside and outside of coding regions (data not shown).

In addition to overall concordance, we examined the false positive rate for each sample and genotype caller, defined as the fraction of variant sites for that sample called with at least one non-reference allele by the genotype caller that are homozygous reference on the Exome Chip. As seen in Figure 2b, the overall false positive rate for all callers is generally less than 0.03, with only a slight dependence on depth. The median values across sample are 0.02 for multi-sample calling, 0.01 for single-sample calling, and 0.003 for LD-aware calling. Surprisingly, in this low-coverage data, multi-sample calling produces a higher false positive rate than the single sample approach, but both are outperformed by LD-aware calling. Note that real false positive rates in a sequencing experiment will likely be higher than those reported here, since we are restricting the current data set to the well-behaved variants that are part of a genotyping chip.

### Site calling

Imputation uses correlations between nearby variants to help call genotypes. Decreased variant frequency causes uncertainty in these correlations and therefore lower fidelity of calling. To investigate the role of variant frequency, we examine the fraction of variant sites discovered by Thunder as a function of frequency and compare with the variant sites discovered by multi-sample Unified Genotyper. We define a variant site as a site at which at least one alternate allele is detected from among all samples in the exome chip data. We consider that site to be found by a variant caller if at least one alternate allele is called at that position, regardless of whether or not any genotypes match.

**Figure 3:**
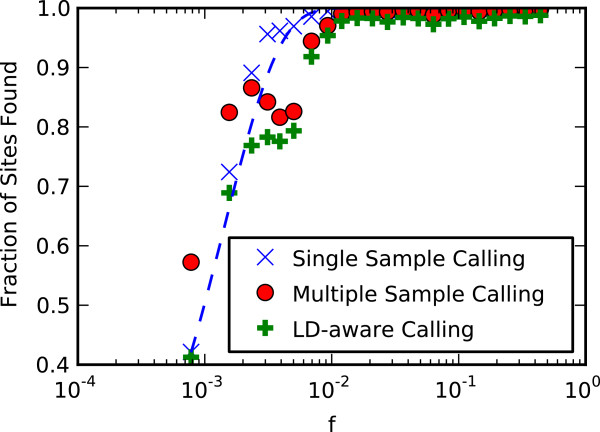
**Frequency dependence of site finding.** The fraction of variant sites found is dependent on both the frequency range of the variant, and the method used to call variants. The GATK Unified Genotyper in multisample mode finds more variants at all frequency ranges, but the disparity is most pronounced at the lowest frequencies, where the Unified Genotyper finds approximately 50% more variant sites than THUNDER. Single-sample Unified Genotyper calls follow a model that assumes a constant probability of finding any site in a single sample.

Figure 3 displays the fraction of sites discovered within a set of frequency ranges. For variant frequencies greater than 0*.*01, both multi-sample Unified Genotyper and Thunder perform well, finding greater than 97% of the variant sites, with slightly more variants being discovered by Unified Genotyper. As the frequency decreases, both methods degrade in performance, however, Thunder degrades more quickly. At the lowest frequencies, where the minor allele count in the exome chip data is 1, Unified Genotyper identifies approximately 57% of the variant sites, while Thunder identifies only 41%. Note that there is no contradiction between higher concordance rates for Thunder and better variant finding with Unified Genotyper, because LD-based methods are less adept at finding variants with a lower minor allele frequency. When a site is not noted as variant by Thunder, this is equivalent to a reference call for all samples. Because these variants have preferentially low frequencies, most of these reference calls will be correct, and the concordance is largely unaffected.

When Unified Genotyper is run in single-sample mode, the probability of finding each variant in a given sample is independent of the frequency of that variant in other samples. The overall probability of finding a site, then, will be increased for higher frequency sites since each sample with the variant provides an independent chance of finding the site. Assuming that the probability of finding any given site in a single sample is *p*, and that the frequency of the variant site is *f*, the probability of finding the site in the cohort is given by *1-(1-p)*^*f*^. A curve of this form is shown in Figure 3, and the single-sample results follow its general shape.

### Confounding effect of kinship

To provide preliminary evaluations of the utility of the called variant data obtained from low-coverage WGS, we attempted to estimate kinship coefficients between all pairs of individuals in the present sample using the called variant data and to generate allele frequencies for the identified loci.

The ability of LD aware genotype calling methods to reliably impute genotypes will be affected by the presence of closely related relatives and the effective population size. This study has both closely related and cryptically related individuals from a Native American tribe as well as population admixture with individuals of known European ancestry. Inclusion of close relatives should improve the ability of LD aware imputation algorithms to call genotypes on specific haplotypes. In contrast, we would expect that the level of admixture present in the study population would effectively reduce the ability of LD aware algorithms to call genotypes. Thus, there is the potential that the results from the present study may not be generalizable to other populations. Because correlations between array generated genotypes and imputed genotypes from sequence data appear to be stable as a function of coverage, the confounding inclusion of relatives and admixture do not appear to be a significant factor. An analysis of a nearly-unrelated subset of the data would not be directly comparable to our original results, because of the large number of samples that must be removed even to limit relations to cousin-level.

**Figure 4:**
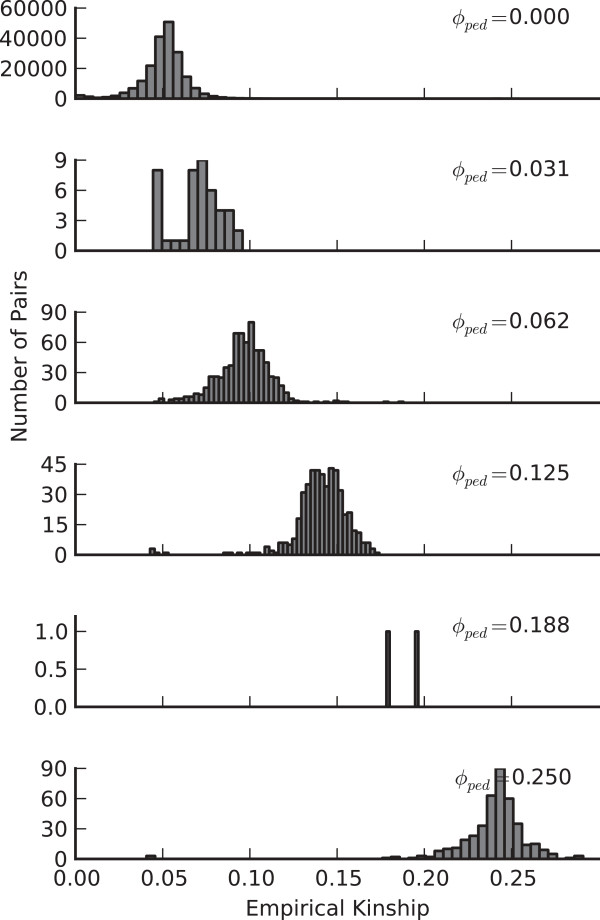
**Empirical kinship coefficents.** Histograms of empirical kinship coefficients calculated from THUNDER genotypes. Each row contains all pairwise values that have the noted value for the pedigree-defined kinship coefficient. Thus, the lowest histogram ( φ_ped_ = 0.25) contains all full sibling and parent–child relations, the next row up contains grandparent-grandchild, avuncular, and half-sibling relations, and so on.

During the process of confirming the relationships between subjects and their self-reported familial relationships, we observed a trend that suggests a possible impact of including subjects with different degrees of relatedness. Figure 4 shows the distribution of kinship coefficients calculated from imputed genotypes using PREST [15] for groups that are expected to have the same kinship coefficient. There is a trend that the measured kinship coefficient is less than expected for closely related individual and greater than expected for distantly related and unrelated individuals. This suggests an uncorrected bias in how genotypes are imputed and/or that individuals have cryptic relationships. A comparison with kinship coefficients calculated from genotypes for a set of common variants contained on the exome chip exhibited greater than expected IBD sharing for all relative pairs suggesting an influence of cryptic relationships (data not shown). The greater than expected IBD sharing for close relative pairs seen in the exome chip genotypes also suggests the possibility of bias in how genotypes are imputed, but this could also be the result of differences in content between the selected set of variants from the WGS dataset and the variants contained on the exome chip that were used to estimate kinship. In either circumstance, the high concordance rates between the WGS and exome chip data indicates this bias has a minimal impact on individual genotype calls.

### Corrected allele frequencies

To estimate allele frequencies for this population, it is necessary to correct for family structure. Simply counting prevalence of alleles will result in an upward bias due to inclusion of alleles that are identical by descent. One solution is measuring the prevalence in a subset of the samples chosen with all pairs having a kinship coefficient calculated from the pedigree structure below a specified threshold. Using a subset of less related individuals, however, will increase error due to sampling from a smaller population. A more powerful solution involves the use of the Best Linear Unbiased Estimate (BLUE) of the allele frequencies based on the observed alleles, and kinship coefficients based on the pedigree structure [16]. Allele frequencies were estimated using this method as implemented in the package MQLS [17]. In the current data set, MQLS-calculated corrections to the simple prevalence frequencies are small, with a root-mean-squared correction across all variants of less than 1%. These corrections are of similar direction and magnitude to those calculated in a simple allele prevalence on a less related subset of the cohort (kinship coefficient less than 0.1). The deviation between these frequencies and the whole sample uncorrected frequencies correlates with the MQLS corrections at *r* =0*.*65 indicating that MQLS approach is performing as expected. This also provides further evidence that any bias in genotype as suggested by the underestimation of IBD sharing among closely related individuals using WGS data is likely to have a minimal impact on genotype calls.

**Figure 5:**
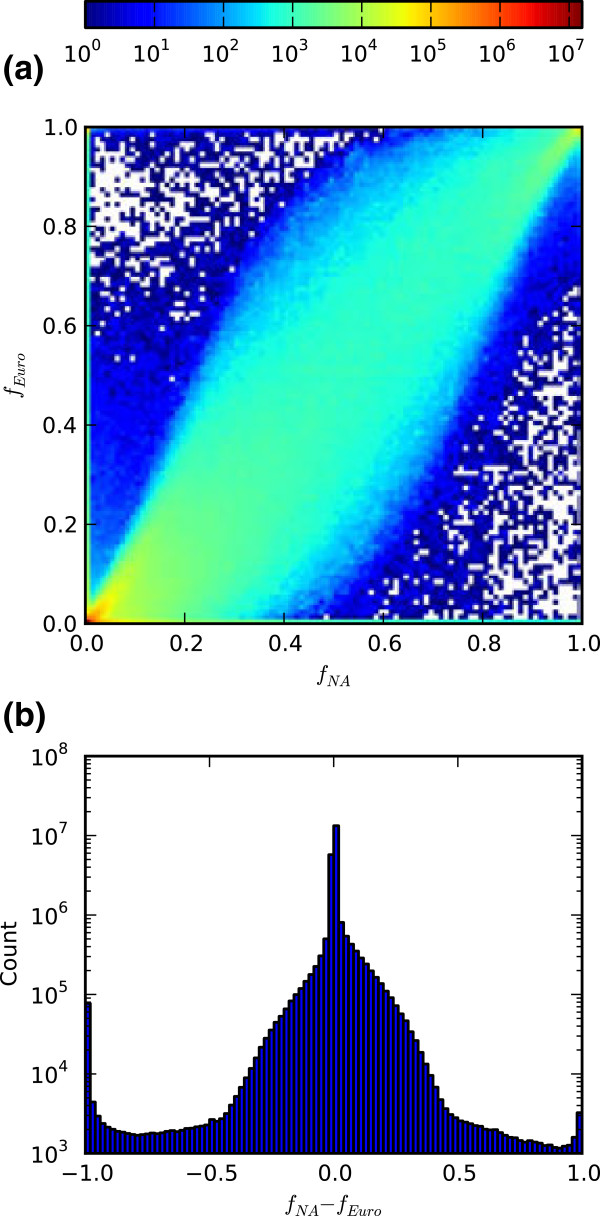
**Allele frequencies in the NA Cohort. a)**. A two dimensional histogram comparing allele frequencies in the Native American cohort with those in European ancestry samples from 1000 genomes. The variants shown are the union of the two sets. Color scales logarithmically with the number of variants as in the colorbar above the image. **b)**. One dimensional histogram of the difference in allele frequency for the same variants as shown in **(a)**.

Figure 5a displays a two-dimensional histogram comparing MQLS-calculated allele frequencies versus the frequencies from 1000 genomes for samples with European ancestry [18]. Figure 5b shows a histogram of the frequency differences between the two populations. In each case, the frequencies displayed are those of the non-reference allele rather than the minor allele. In each figure, similar features can be observed. First, the distribution is strongly peaked, mostly at very lopsided allele frequencies, where the European and Native American samples agree closely. Second, the distribution has wide tails of highly-variant allele frequencies. Third, there is asymmetry between the two populations with a greater number of variants having a higher frequency in the European-ancestry cohort than in the Native American group, reflecting the admixture of the current Native American data set.

## Conclusions

Low-coverage WGS represents a genotype sampling strategy between fixed content SNP arrays and deep WGS, but there are economic and coverage trade-offs between each of these approaches. Because fixed content SNP arrays became economically viable before either of the sequencing approaches, much has been written about the proportion of common sequence variants that can be imputed with commonly used, fixed content SNP arrays (e.g. [19]). Currently, however, there is substantial interest in studying increasingly rare variants in association studies of complex traits, which has led to increased focus on WGS approaches. Assuming that this is likely to continue and the costs associated with WGS will continue to drop, WGS will eventually prevail as the analysis method of choice leading to the question of whether to pursue low-coverage or deep WGS strategies.

Depth of coverage and calling technology are not chosen independently when designing a sequencing experiment. Rather, an experimenter with a fixed budget must choose between fewer samples with higher coverage and more samples with lower coverage. The ability of LD-based calling makes the latter option viable, but previous studies do not explicitly show it to be preferable.

To make clear the viability of low-coverage sequencing, the present study evaluated variant calls from low-coverage sequence data using the LD-aware calling software Thunder and the single- and multi-sample calling options in Unified Genotyper. The results demonstrated increased fidelity of variants calls made using the LD-aware Thunder relative to calls made using multi-sample GATK to genotypes generated from a fixed content SNP microarray. Nonetheless, this increased fidelity came at the cost of failing to identify a number of very low frequency variants (i.e., <0.5%). Despite this trade-off, we conclude that low-coverage sequencing still presents specific advantages over deep sequencing when economic conditions are fixed. These advantages are best illustrated using a hypothetical example in which we assume that equivalent genotype concordance could be achieved with LD-aware calling at 5x coverage and multi-sample calling at 15x coverage as suggested by Figure 2. In such a case, as seen in Figure 3, the call rate of the lowest frequency variants will be approximately 40%. However, for a fixed cost, three times more samples can be sequenced at low coverage than at high coverage. Even assuming, in a worst case, that the LD-aware call rate will still be 40%, and further assuming that multi-sample variant calling at 15× coverage results in 100% call rate for the lowest-frequency variants, the total number of low-frequency variants discovered will still be greater in the lower coverage sample. This advantage becomes even more apparent if the higher-coverage sample is sequenced at a depth of 30× or greater.

Furthermore, the net effect of the ability to find rare variants is unlikely to be felt in single-variant association tests, where it is uncommon for sample sizes to be sufficient to detect a significant association. Rather, burden tests, in which attempt to identify genes or pathways where more deleterious mutations are observed in cases than controls (e.g. [20,21]) are expected to benefit from the greater discover of rare variants.

In the present study, we have not attempted to compare these variant callers across a full range of cohort sizes and depths, and cannot therefore generalize that any set of parameters will produce similar results using LD-aware calling. However, the performance of LD-aware calling as a function of these parameters has been addressed via extensive simulations in [9], revealing several trends. First, genotype concordance is roughly independent of sample size (above 30) for coverage above 6X. Second, as coverage decreases, the concordance decreases for smaller cohorts; at 2X coverage, 200 samples are required to produce concordance equivalent to the 6X case. Finally, the SNP discovery rate is far more sensitive to cohort size than concordance.

To provide a preliminary demonstration of the utility of the called variant data obtained from low-coverage WGS, we used these data to confirm the kinship structure of the study pedigrees, and compared calculated founder allele frequencies for this sample to those reported in the 1000 genomes project for European ancestry participants. Though this would represent only a very preliminary use of low-coverage WGS data in a gene-finding expedition, this illustration suggests that these data are appropriate for further analysis in linkage and associations studies.

Though secondary to scientific concerns, the costs of storage and computation must also be considered when choosing sequencing and variant calling strategies. Storage costs do not actively influence the choice of high or low coverage WGS because the storage scales with total number of reads, while the choice between high and low coverage is whether those reads will be distributed across few or many samples. The aligned sequence read data for this project with 708 samples with an average read depth of 7.9 was 21 terabytes. In a high-coverage sequencing project with the same sequencing budget, the storage would be approximately the same, but fewer samples would be covered. The storage of variant data that is used by each approach for association analysis is roughly proportional to the product of the sample size and number of variants typed or imputed, and it is typically negligible compared to the read data. In this data set the compressed VCF files with 708 samples and 21.7 M variants require 65 gigabytes or 4.23 bytes per genotype, while the exome chip data in binary plink format for 730 samples for 225115 SNPs require 46 megabytes or 0.28 bytes per genotype. The main difference between the per-genotype size of the data files is the amount of stored supporting information and compression.

There is also a substantial greater cost required for the infrastructure required by both low-coverage and deep WGS analysis than fixed content genotype array analysis. The infrastructure for imputation with low-coverage WGS adds substantially to the cost. In this experiment the average cpu time for producing all-site single sample GATK Unified genotyper calls was 32 hours using 4 cores of a single node in a linux cluster with 122 blade servers, each with 8-core 2.80 GHz Intel processors, 2×4 M L2 cache (Model X5560) and 48 GB of shared memory. The same cluster used approximately 25000 CPU hours to run Thunder on all 708 samples. More than 300 Terabytes of disk space would have been used had metafiles generated during processing not been regularly deleted.

Two aspects of Thunder may limit its use in some situations. First, Thunder only operates on SNPs, and does not call indels. Second, Thunder only works on autosomal chromosomes. Neither of these limitations is fundamental in nature, but overcoming each will require further development. Because of the advantages of low-coverage WGS described above it is likely that it will increasingly be used because of its economic advantages over deep WGS.

The present report demonstrates the viability of low-coverage WGS for identifying variants associated with complex traits and describes specific advantages this approach might possess over deep WGS. Notably, additional improvements in variant calling routines are likely to occur, which could further improve data generated from low-coverage WGS. For example, while LD-based calling improves the overall call quality of low-coverage sequencing, low-coverage does not preclude the use of multi-sample techniques to discover low-frequency variant sites. Though we have not explored this possibility, an approach merging multi-sample results at low frequency with LD-based results at a higher frequency may be better than either method alone. Because LD-aware calling relies on correlations of nearby variants, its performance may be enhanced in a data set with many related samples, as in the current study. However, the performance of LD-based calling in settings with unrelated data [9] suggests that this effect is not vital to the conclusions presented here.

## Methods

### Participants

The protocol for the study was approved by the Scripps Institutional Internal Review board and Indian Health Council, a tribal review group overseeing health issues for the reservations where recruitments took place. Written informed consent was obtained from each participant after study procedures had been fully explained. Participants were compensated for their time spent in the study. Participants who were of Native American Heritage were targeted for study and recruited from eight geographically contiguous reservations with a total population of about 3,000 individuals. Participants who were mobile and between the ages of 18–82 years were recruited using a combination of a venue-based method for sampling hard-to-reach populations [22,23] and a respondent-driven procedure [24], as reported previously [25]. Demographic characteristics of this Native American population have been reported previously [25].

### Exome chip genotyping

Previously isolated DNA samples were measured for quality control and concentration using a Nanodrop spectrophotometer. Quality control metrics suggested by the Affymetrix Axiom 2.0 Assay Manual Workflow documentation were adopted and applied. Samples were expected to achieve an *A*260*/*280 between 1.8 and 2.2, an *A*260*/*230 greater than 1.5, and a measured concentration 50 ng/μl or greater. Samples that passed these quality control metrics were randomly assigned to 96-well plate positions and diluted to 50 ng/μl; each plate included an Affymetrix-provided internal control gDNA sample at this same concentration. All plates were stored at −20°C until submitted for fragmentation, hybridization, labeling, and scanning. The Axiom 2.0 Assay Automated Workflow User Guide was followed to prepare all DNA samples for genotyping. The Beckman Coulter Biomek FXP Automated Laboratory Workstation and Affymetrix GeneTitan MC Instrument were used for all sample preparation, hybridization, ligation, washing, staining and scanning of the samples. Briefly, 200 ng of gDNA is amplified for 23 hours at 37°C using Module 1 of the Axiom 2.0 Reagent Kit. After amplification, the samples are fragmented using Module 2 of the Axiom Reagent Kit. The fragmented DNA is precipitated overnight at −20°C. The precipitated DNA is centrifuged for 40 min at 4°C at 3200 × g (4000 RPM) in an Eppendorf 5810R centrifuge. Re-suspension and hybridization preparation of the sample is carried out using Module 2 of the Axiom Reagent Kit. Following preparation of the hybridization plate, the samples are denatured and transferred to a GeneTitan hybridization tray. The Axiom array plate and hybridization tray are then loaded onto the GeneTitan MC Instrument. The samples hybridize on the GeneTitan for 23.5 hours. Following hybridization, ligation and stain trays are prepared and loaded onto the GeneTitan MC Instrument. Ligation, washing, staining and scanning of the array is carried out on GeneTitan MC Instrument. Initial sample and array quality is assessed using the Affymetrix Genotyping Console Software. Variants are initially subjected to Affymetrix quality control as described by Affymetrix [26]. A control sample was included on all plates. Additionally, we ran 56 samples in duplicate; any variant with discordant results across more than three of these pairs was also removed. Hardy-Weinberg p-values were calculated using plink [27,28] on a subset of 239 unrelated samples, and those variants with a *p <* 10^-10^ were removed. Finally, three samples were removed that had an apparent discordance between reported gender and gender as calculated using plink.

### Whole genome sequencing

The DNA libraries were prepared according to Illumina TruSeq™DNA Sample Prep protocol. Total cellular DNA was sheared to sizes between 200 and 800 base pairs by using a Covaris S2 sonicator according to the Illumina TruSeq DNA protocol and then libraries were prepared using the Illumina TruSeq™DNA Sample Prep Kit on Tecan Freedom EVO 200 Automated Liquid Handling System. Libraries were size selected for insert fragments around 300 base pairs using Pippin Prep automatic DNA size selection system (Sage Science). Libraries were analyzed and quantified using a LabChip GX automated electrophoresis system (Caliper) and diluted to 15 nM concentration. The paired-end sequencing (2 × 100 cycles) was performed on HiSeq2000 sequencers (Illumina). Initially each library was sequenced on a single lane of a flow cell. The measured concentration of DNA in a library is an unreliable predictor of the optimal amount of DNA that should be loaded on a flow cell. During the course of this experiment we converted to a strategy where 24 samples were pooled and run on multiple lanes of a flow cell such that the optimal amount of DNA could be determined to enable collection of the greatest amount of quality sequence per lane.

### Sequencing metrics

We define depth of coverage in WGS as the number of mapped bases divided by the total length of the reference genome; Figure 1 displays the distribution of sample coverage. The central 80% of the samples are approximately evenly distributed between coverage of 3X and 12X. As basic quality control metrics, we examine the percent of reads that map to the genome (median 91.925%), and the percent that are correctly paired (median 88.325%).

### Variant calling

Reads from whole genome sequencing were aligned using BWA version 0.5.8c (each of the two fastq files is used to create a sai file using bwa aln –t 4, then these files are combined with bwa sampe –p illumina). Picard 1.48 was used to de-duplicate and sort the resulting BAM files. GATK v1.0.5777 was used to realign near indels (−et NO_ET –dt NONE). Following Picard FixMateInformation, GATK was used to recalibrate BAM quality scores using the –standard_covs argument to CountCovariates (so that the bam is reclibrated on ReadGropu, QualityScore, Cycle, and Dinuc). For samples that ran on more than one lane, we performed these calculations on each lane individually. We calculated variant calls independently for each BAM file using GATK Unified Genotyper and following the best-practices for low-coverage samples [7,29]. To guard against sample misidentification, we compared these initial variant calls to the results of the exome chip genotyping. Given an arbitrary sample’s exome chip results, and another sample’s WGS variant calling, we define a quantity *M*

$$M\equiv \prod_{\text{markers}}\frac{L\left\{\left(D\right|GT_{\mathrm{EC}}\right\}}{f_{\mathrm{GT}}}$$

where *L*{*D*|*GT*_*EC*_ }, the likelihood of the sequencing data given the exome chip genotype is calculated by the Unified Genotyper, and recorded in its PL annotation, and *f*_*GT*_ is the frequency of the exome chip genotype, as estimated from the exome chip data. This quantity will be maximized when the two samples are identical. We calculate *M* for each pair of samples. Cases in which *M* was maximized for an apparent mismatch were further investigated using calculated kinship coefficients compared to the known pedigree structure. In cases where apparent mismatches could not be resolved, samples were removed from analysis. For the samples that ran on more than a single lane, BAM files were combined to produce a single BAM file for each sample. This set of files was used to perform three types of variant calling. First, single sample variant calls were made using Unified Genotyper v2.1-8 with low-coverage parameters (−stand_call_conf 4 –stand_emit_conf 0 –dcov 250). Second, multi-sample variant calling across all samples, using Unified Genotyper with the same parameters as the single-sample call. Third, LD-aware variant calls were calculated in a multi-stage process that initially creates genotype-likelihood files (GLF) for single samples using samtools-hybrid [30,31], creates initial haplotypes with BEAGLE [32], and then runs Thunder using the BEAGLE haplotypes as input. Specifically, samtools-hybrid is used to create single-sample GLFs. This call is parallelized by calling each region of 5 million base pairs separately. The glfMultiples tool combines all of the sample GLFs for one region into a single VCF. The regions are combined into per-chromsome multi-sample VCFs, and variants are filtered to include only passing variants using a combination of infoCollctor and vcfCooker. Each single-chromosome multi-sample VCF is split into groups of 10000 variants, with an overlap of 1000 variants. These groups are each run through BEAGLE for 50 iterations, and then re-attached to chromosomal VCFs using ligateVCF. Again, the chromosomal VCFs are split into groups of 10000 variants with an overlap of 1000. Each of these VCFs (containing all samples) is used as input to thunder, and the resulting phased VCF files are again joined at the chromosome scale using ligateVCF. This pipeline is run with a version of UMAKE [33] that has been modified to fit the local computing environment. The distribution of UMAKE also contains the intermediary scripts mentioned above, such as vcfCooker, and ligateVCF. At no time in the process is pedigree information of the samples taken into account.

This variant calling activity was performed on 708 samples. However, only a subset of the samples had measured genotypes using the Axiom Exome Chip, so the final comparisons were carried out using the 641 samples in common.

### Confounding effects of kinship and allele frequency correction

Kinship coefficients for all pairwise individuals were generated from exome chip genotypes and sequence data were estimated using PREST-plus v4.09 [15].

For calculation of the allele frequencies given the pedigree information, initially KinInbcoef v1.1 is used to create kinship coefficients from the known pedigree structure in the proper format for MQLS. Then MQLS v1.5 is used with option 1, and all samples with unknown phenotype. With MQLS option 1, the unphenoytped samples are used in the estimate of the allele frequency.

## Competing interests

The authors declare that they have no competing interests.

## Authors’ contributions

CE and KW designed the research program. CE collected all DNA samples and pedigree/admixture information. SC carried out exome chip data collection. EM and PM performed the WGS. Data was analyzed by XW, JS, MS, IG, and CB. YL provided expertise on variant calling. CB, IG, KW, and CE were involved in manuscript preparation. All authors read and approve the final manuscript.
